# Comparative analysis of proso millet (*Panicum miliaceum* L.) leaf transcriptomes for insight into drought tolerance mechanisms

**DOI:** 10.1186/s12870-019-2001-x

**Published:** 2019-09-11

**Authors:** Yuyu Zhang, Xiaoli Gao, Jing Li, Xiangwei Gong, Pu Yang, Jinfeng Gao, Pengke Wang, Baili Feng

**Affiliations:** College of Agronomy, Northwest A&F University / State Key Laboratory of Crop Stress Biology in Arid Areas, Yangling, 712100 Shaanxi China

**Keywords:** Drought, Proso millet, RNA-seq, Gene expression, Jasmonic acid

## Abstract

**Background:**

Drought stress is a major abiotic stress that causes huge losses in agricultural production. Proso millet (*Panicum miliaceum* L.) can efficiently adapt to drought stress and provides important information and gene resources to improve drought tolerance. However, its complex drought-responsive mechanisms remain unclear.

**Results:**

Among 37 core Chinese proso millet cultivars, Jinshu 6 (JS6) was selected as the drought-sensitive test material, whereas Neimi 5 (NM5) was selected as the drought-tolerant test material under PEG-induced water stress. After sequencing, 1695 differentially expressed genes (DEGs) were observed in JS6 and NM5 without PEG-induced water stress (JS6CK and NM5CK). A total of 833 and 2166 DEGs were found in the two cultivars under simulated drought by using 20% PEG-6000 for 6 (JS6T6 and NM5T6) and 24 h (JS6T24 and NM5T24), respectively. The DEGs in JS6T6 and JS6T24 treatments were approximately 0.298- and 0.754-fold higher than those in NM5T6 and NM5T24, respectively. Compared with the respective controls, more DEGs were found in T6 treatments than in T24 treatments. A delay in the transcriptional responses of the ROS scavenging system to simulated drought treatment and relatively easy recovery of the expression of photosynthesis-associated genes were observed in NM5. Compared with JS6, different regulation strategies were observed in the jasmonic acid (JA) signal transduction pathway of NM5.

**Conclusion:**

Under PEG-induced water stress, NM5 maintained highly stable gene expression levels. Compared with drought-sensitive cultivars, the different regulation strategies in the JA signal transduction pathway in drought-tolerant cultivars may be one of the driving forces underlying drought stress tolerance.

**Electronic supplementary material:**

The online version of this article (10.1186/s12870-019-2001-x) contains supplementary material, which is available to authorized users.

## Background

Drought stress is a chronic, random, and unpredictable abiotic stress that causes huge losses in agricultural crop production [1–3]. Drought was considered a major cause of severe food shortages and famine [2]. Present statistical data have revealed that 40% of the world’s agricultural production comes from irrigated lands, which accounts for 70% of the world’s water reservoir [4]. It remains a major limitation of agricultural production. With the increase in world population and global warming, drought stress will exacerbate in the future due to limited water resources [5–7]. Therefore, to improve agricultural production, drought tolerance mechanisms must be understood.

With satisfactory adaptation to drought, high temperature, and poor soil conditions, proso millet (*Panicum miliaceum* L.) is important in tasks related to drought resistance [8–10]. It is a C_4_ crop and tetraploid species (2 *N* = 4X = 36) with a short growing season [11]. Proso millet is one of the oldest cultivated cereals with great historical significance [12, 13] and is mainly planted as a source of staple food and fodder in the semi-arid regions of China [14, 15]. It is also planted as a makeup crop in case of major crop failure [15]. Therefore, insights into the drought tolerance mechanisms and the important genetic resources of proso millet will help improve its drought tolerance and that of other crops.

Drought-responsive mechanisms, including various molecular, morphological, and physiological responses, help plants survive drought stress and maintain productivity. Plants have morphological and physiological responses, including improvement of root traits [16], reduction of epidermal (stomatal and cuticular) conductance and decrease in evaporative surface (leaf area) and radiation absorption [3], to cope with water-deficit conditions. Hormones regulate the complex network of signal transduction to adjust and control plant growth in stress-free situations and survive under stress [3]. When plants experience stress, the first step involves perception of environmental signals [17]. In the presence of abiotic stresses, abscisic acid (ABA), an important plant hormonal signal, provides one of the fastest responses and triggers subsequent defences [18]. It is responsible for regulating stomatal closure [19], which is the primary way of saving water via gaseous exchange in plant leaves. In response to abiotic stress, the complex cross talk among different plant hormones causes synergetic or antagonistic effects [20]. ABA and jasmonic acid (JA) have played positive roles in regulating stomatal closure, whereas auxins and cytokinins have played negative roles [3, 21]. Moreover, the role of ethylene is determined by different tissues under varying conditions [3].

JA plays important roles in stress response by regulating the balance of plant growth and defence response and enabling plants to adapt to changing conditions [22–24]. Water stress induces JA accumulation to promote the synthesis of ascorbate peroxidase, monodehydroascorbate reductase, glutathione reductase, dehydroascorbate reductase, ascorbic acid, and glutathione [25]. The ascorbate–glutathione cycle in plant cells highly contributes to scavenging reactive oxygen species (ROS) [26]. This cycle works in all the organelles where ROS detoxification is needed [27, 28]. According to Bartoli et al. [29], the abundance of ascorbate is responsible for the regulation of cellular redox buffering capacity, which influences the threshold of activating hormone signals and the interactions between different hormones. In addition, apoplastic ascorbate helps alleviate the enhanced oxidative burden from stresses and is the first line of defence against potentially damaging external oxidants [30].

Under normal conditions, ROS are inevitable products of aerobic metabolic processes [31–33], including photosynthesis and respiration; ROS levels are usually low and can be strictly controlled [26]. However, water stress can disrupt cellular homeostasis and increase ROS production, thereby triggering specific oxidative responses [26]. Under stress, ROS are secondary messengers in signalling cascades that convey important information to regulate hormonal changes in concentrations and/or sensitivity to activate downstream stress responses and defence processes [29]. However, ROS are overgenerated during drought stress [31–33] and can oxidize key cellular components and cause oxidative cell destruction [28, 34]. Moreover, overgeneration of ROS is fatal to protein, membrane, and DNA and can cause cell death [35–37], thereby posing a major threat to plant cells. Superoxide dismutase (SOD), catalase (CAT), ascorbate, glutathione, and peroxidase (POD) are also involved in various defence responses [2, 38] and play key roles in ROS scavenging under biotic and abiotic stresses [39].

Under drought conditions, genes regulate the pathways related to osmoprotectants to directly protect important proteins and membranes [40]. Adverse conditions can induce the responses of membrane transporters and ion channels related to water and ion uptake [41]. These adverse conditions can induce related transcription factors that regulate the downstream stress-related genes to cope with water stress [2]. Knowledge of genomic information is crucial to understand the molecular mechanism of drought tolerance and provide important gene resources. Recently, RNA sequencing (RNA-seq), which is widely used in various plants, has proven to be a highly efficient method to achieve transcriptome data [42–44]. However, previous studies on drought tolerance of proso millet mainly focused on its morphological and physiological responses [45]. In recent years, several studies [13, 45] have reported the transcriptome characteristics of proso millet. However, the mechanism underlying leaf response and adaptation to drought through changes at the molecular level remains unclear, particularly among different drought-tolerant cultivars.

In the present study, to further elucidate the molecular basis of the response of proso millet leaves to drought stress and the drought tolerance mechanisms of different drought-tolerant cultivars, we selected drought-sensitive and drought-tolerant cultivars. Their transcriptome was analyzed based on RNA-seq data. Our results will provide information that can help improve our understanding of the molecular mechanism underlying drought tolerance. Moreover, this study provides important gene resources for improving the tolerance of proso millet and other crops.

## Results

### Evaluation of the drought tolerance of proso millet cultivars

Among the 37 cultivars, the mean germination rate of the control group was approximately 92.3%, and their highest and lowest germination rates were 99.0 and 74.0%, respectively. The mean germination rate of the simulated drought treatment group was 72.9%, and their highest and lowest germination rates 92.3 and 43.0%, respectively. The mean relative germination rate was 79.0%, and the highest and lowest relative germination rates were 101.6 and 46.6%, respectively (Additional file 1: Table S2). The germination rates of the control group exceeded 95%, and the highest and lowest relative germination rates of the simulated drought treatment group were 96.9 and 48.6%, respectively. Among these cultivars, Neimi 5 and Jinshu 6 (renamed NM5 and JS6, respectively) were selected as drought-tolerant and drought-sensitive test materials for subsequent analysis, respectively.

### Differences in malondialdehyde (MDA) contents in NM5 and JS6 under PEG-induced water stress

**Fig. 1:**
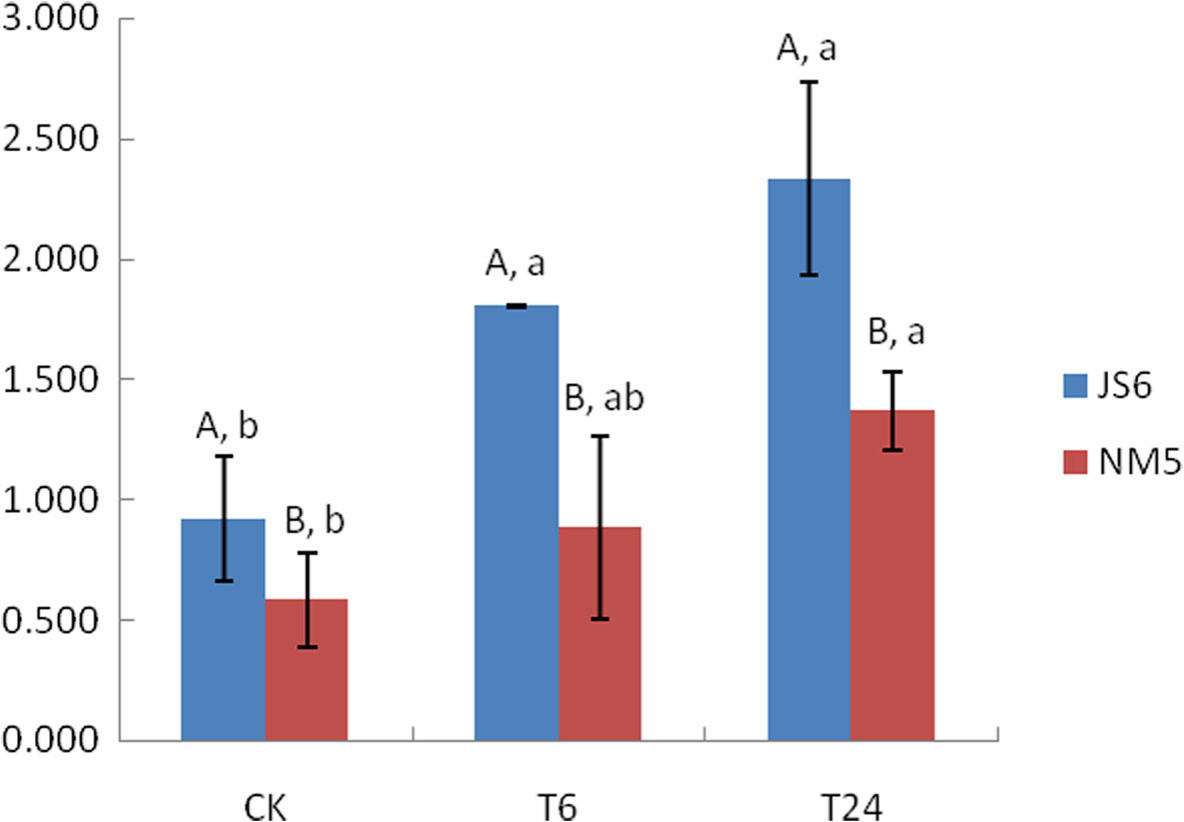
MDA content changes in the leaves of JS6 and NM5. JS6 and NM5 plants at the three-leaf stage were treated with 20% PEG-6000 solution for 6 h (JS6T6 and NM5T6, respectively), 24 h (JS6T24 and NM5T24, respectively), or kept in water (control). After treatments, leaf tissues from each group were sampled for MDA measurement. All data are shown as mean ± standard error. Different uppercase letters represent significant differences between cultivars; different lowercase letters indicate significant differences between treatments (Duncan test; *P* < 0.05)

In the control group, JS6 and NM5 were designated as JS6CK and NM5CK, respectively. In the group of 20% PEG-6000 treatment for 6 h, JS6 and NM5 were designated as JS6T6 and NM5T6, respectively. In the group of 20% PEG-6000 treatment for 24 h, JS6 and NM5 were designated as JS6T24 and NM5T24, respectively. MDA contents were elevated in both cultivars as the stress time continued (Fig. 1). JS6 consistently showed higher MDA contents than NM5, regardless of the presence or absence of PEG-induced water stress. Specifically, the MDA contents in JS6 were approximately 0.584-, 1.042-, and 0.706-fold greater than those in NM5 under normal conditions, T6 treatment, and T24 treatment, respectively. Compared with the MDA content under normal conditions, the MDA contents in the JS6 cultivar increased by 95.6 and 152.9% under T6 and T24 treatments, respectively. Compared with the control, the MDA contents in the NM5 cultivar increased by 51.8 and 134.8% under T6 and T24 treatments, respectively.

### RNA sequencing and de novo assembly

Samples for RNA-seq were obtained from 18 libraries of the two cultivars (containing three biological replicates). All the raw reads of RNA sequencing data were deposited in the NCBI Short Read Archive database under the accession number SRP144636 (SAMN08947059–SAMN08947076), as associated with the BioProject PRJNA454008. About 42 million high-quality reads were obtained from each sample (Table 1). A total of 115,660 transcripts and 59,035 unigenes were obtained with a mean length of 1367 bp and N50 of 2044 bp and a mean length of 1080 bp and N50 of 1953 bp, respectively. The transcriptome shotgun assembly data were deposited at DDBJ/EMBL/GenBank under accession number GHHA01000000.

**Table 1:** Information of RNA-seq performed for proso millet leaf tissue in the presence or absence of simulated drought treatment

Sample	Total bases	Total reads	Quality filtered bases (%)	Quality filtered reads (%)
JS6CK-1	6,487,530,000	43,250,200	6,314,524,327(97.33%)	42,131,554(97.41%)
JS6CK-2	6,509,234,400	43,394,896	6,363,855,996(97.77%)	42,458,184(97.84%)
JS6CK-3	6,696,348,000	44,642,320	6,557,437,381(97.93%)	43,748,814(98.00%)
NM5CK-1	6,247,170,600	41,647,804	6,107,549,061(97.77%)	40,747,270(97.84%)
NM5CK-2	6,747,776,700	44,985,178	6,571,843,868(97.39%)	43,846,186(97.47%)
NM5CK-3	5,807,890,200	38,719,268	5,688,608,498(97.95%)	37,951,394(98.02%)
JS6T6–1	6,057,733,500	40,384,890	5,943,426,904(98.11%)	39,651,746(98.18%)
JS6T6–2	7,144,457,400	47,629,716	6,993,164,558(97.88%)	46,654,198(97.95%)
JS6T6–3	7,505,556,300	50,037,042	7,331,461,113(97.68%)	48,914,060(97.76%)
NM5T6–1	6,195,031,200	41,300,208	6,073,088,662(98.03%)	40,518,262(98.11%)
NM5T6–2	5,989,206,900	39,928,046	5,878,371,054(98.15%)	39,216,888(98.22%)
NM5T6–3	6,496,346,700	43,308,978	6,323,637,814(97.34%)	42,190,100(97.42%)
JS6T24–1	5,748,725,700	38,324,838	5,646,195,618(98.22%)	37,667,488(98.28%)
JS6T24–2	7,100,685,000	47,337,900	6,930,777,593(97.61%)	46,242,434(97.69%)
JS6T24–3	6,987,597,600	46,583,984	6,831,757,689(97.77%)	45,579,628(97.84%)
NM5T24–1	6,640,053,600	44,267,024	6,523,588,317(98.25%)	43,521,448(98.32%)
NM5T24–2	5,929,631,400	39,530,876	5,824,098,355(98.22%)	38,854,876(98.29%)
NM5T24–3	5,753,017,800	38,353,452	5,656,189,224(98.32%)	37,733,478(98.38%)
Average	6,446,888,500	42,979,257	6,308,865,335	42,090,445

### Functional annotation

**Fig. 2:**
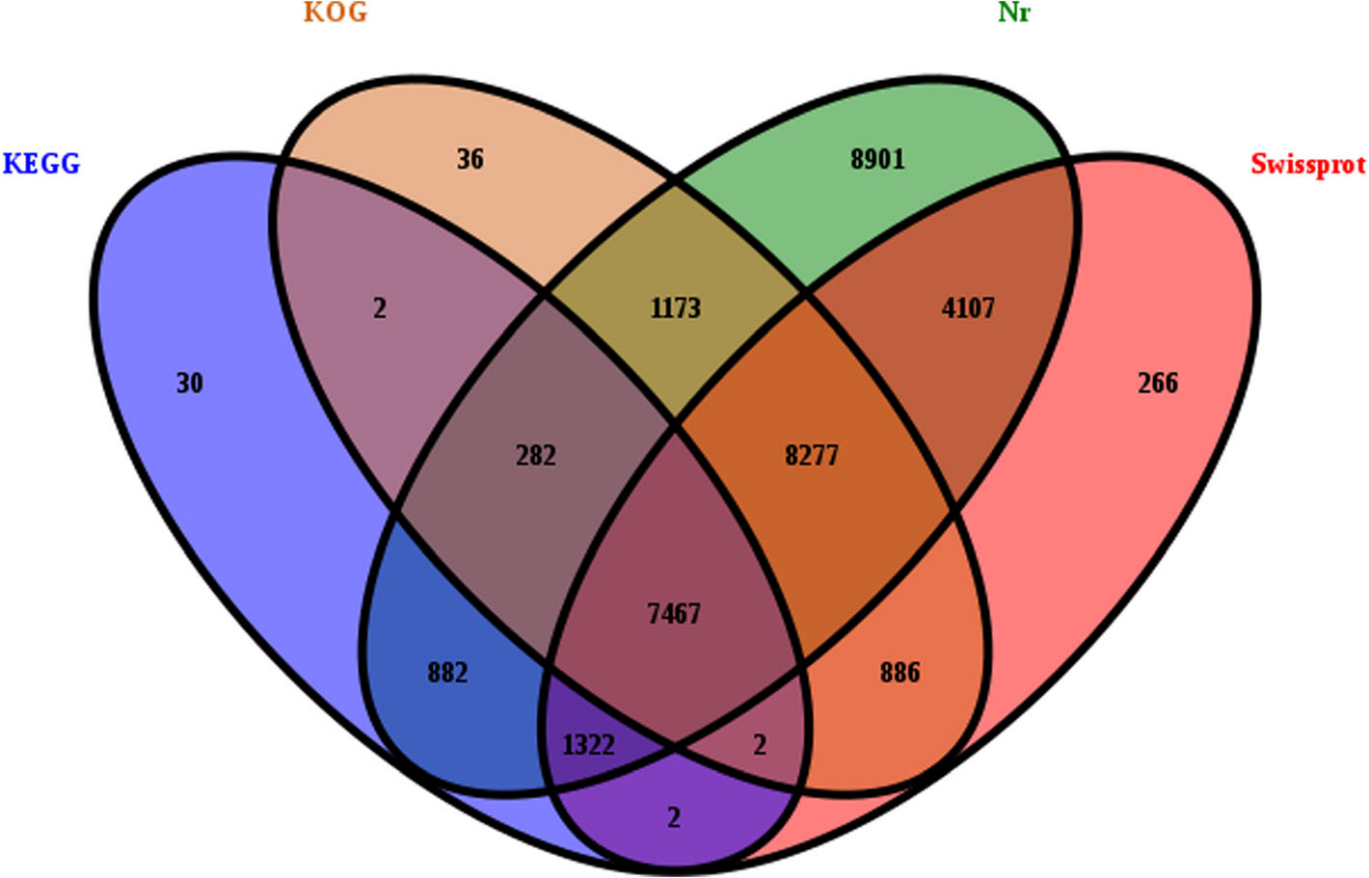
Venn diagram of unigenes annotated by the BLASTX algorithm with an *e*-value <1e^− 5^ in four databases

A total of 33,634 unigenes were annotated in at least one database among the 59,035 unigenes, including 32,410 unigenes annotated in the nr database, 22,328 unigenes in SWISS-PROT, 9988 unigenes in KEGG, and 18,124 unigenes in the COG database (Fig. 2).

KEGG pathway enrichment analysis revealed that 5631 unigenes were annotated in known pathways. The genes related to endocytosis were the most abundant in the cellular process class, and the genes involved in plant hormone signal transduction accounted for the largest part of the environmental information processing class. Genes related to ribosome, spliceosome, protein processing in the endoplasmic reticulum, and RNA transport were highly abundant in the genetic information processing class. Those involved in carbon metabolism and biosynthesis of amino acids accounted for the largest part of the metabolism class, and those related to plant–pathogen interaction were the most abundant in the organismal system class (Additional file 1: Table S3).

**Fig. 3:**
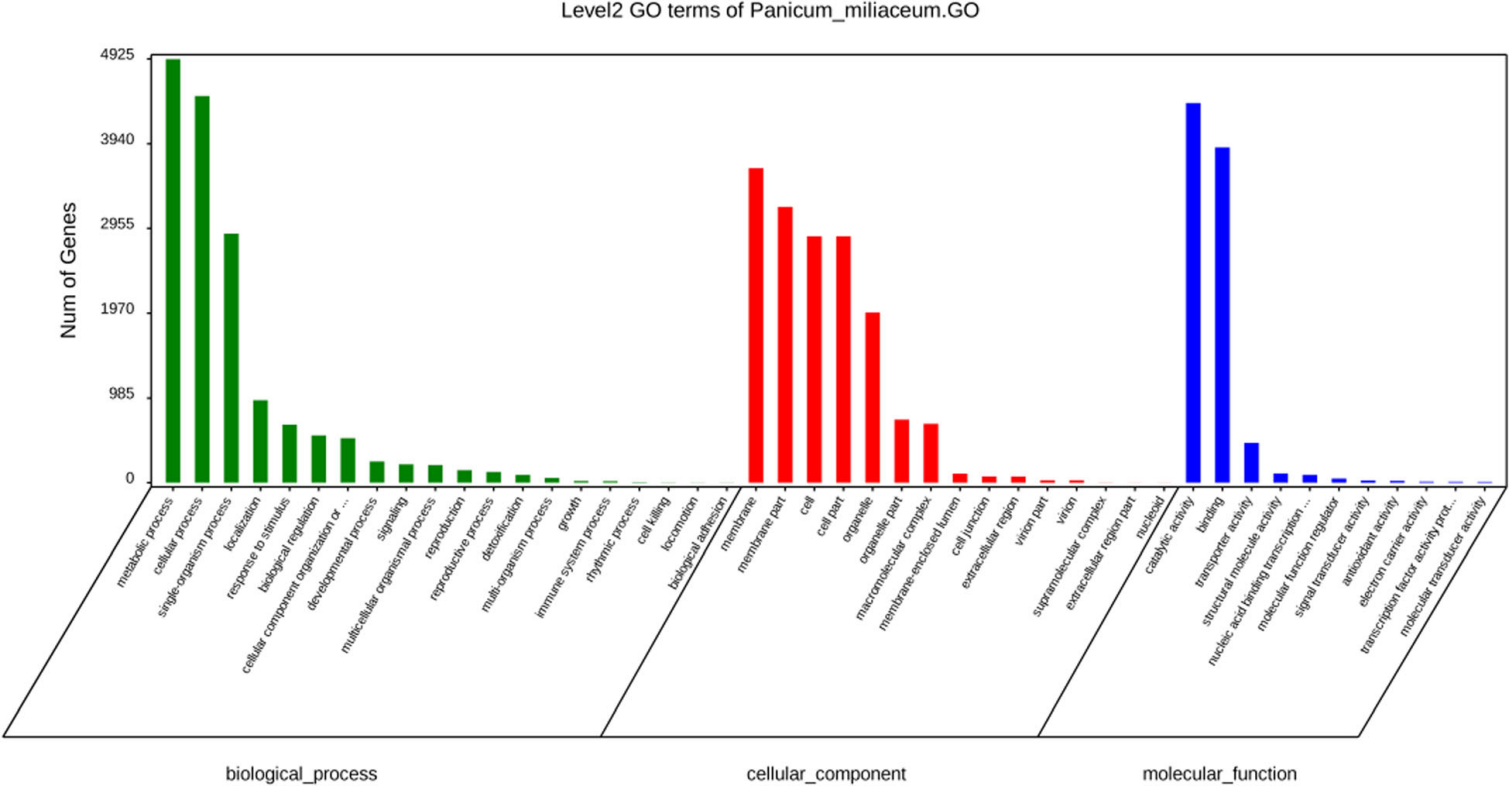
Functional annotations of the assembled unigenes based on GO term analysis. The results are classified into three major GO classes, namely, biological process, cellular component, and molecular function

In accordance with gene ontology (GO) term analysis, 10,085 unigenes were annotated in three major GO classes, namely, 5892 unigenes in cellular component, 6676 unigenes in molecular function, and 6680 unigenes in biological process. The unigenes related to “membrane”, “membrane part”, “cell”, and “cell part” were highly abundant in the cellular component class. In terms of biological process, the unigenes were highly abundant in “metabolic process” and “cellular process” categories, while the genes involved in “catalytic activity” and “binding” accounted for the largest and second largest parts of molecular function class, respectively (Fig. 3).

### Overall analysis of differentially expressed genes (DEGs) in JS6 and NM5 cultivars

**Fig. 4:**
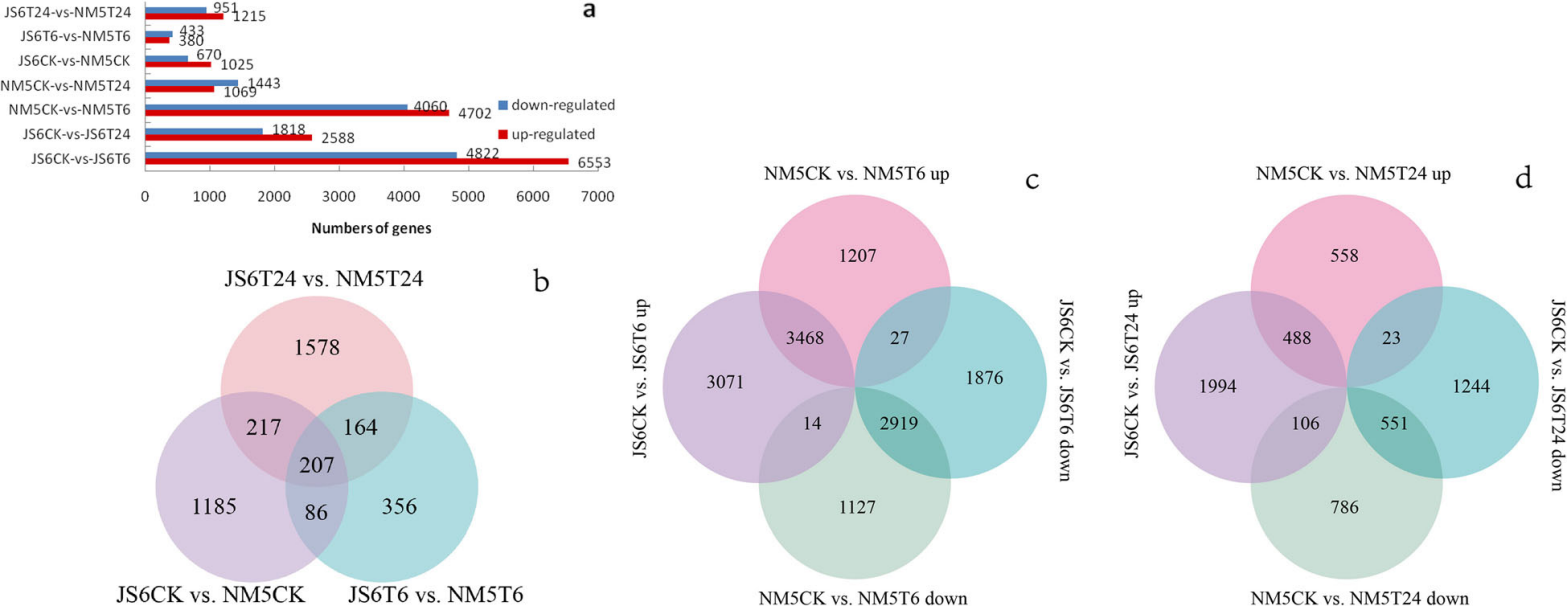
Differentially expressed genes (DEGs) in the presence and absence of simulated drought. DEGs were selected based on a cut-off of FDR ≤ 0.05 and |log2 FC| ≥ 1. **a** Summary of the number of DEGs in the presence and absence of simulated drought. **b** Venn diagram indicating the DEGs from comparisons of JS6CK versus NM5CK, JS6T6 versus NM5T6, and JS6T24 versus NM5T24. **c** Euler diagram of DEGs from comparisons between the control group and T6 treatment, including up- and down-regulated genes in JS6T6 and NM5T6. **d** Euler diagram of DEGs from comparisons between the control group and T24 treatment, including up- and down-regulated genes in JS6T24 and NM5T24

In the control group, 1695 DEGs were observed in JS6 and NM5. Among the samples collected after simulated drought treatment for 6 and 24 h, 833 and 2166 DEGs were identified in JS6 and NM5, respectively (Fig. 4a). The Venn diagram showed that 207 genes were differentially expressed in the two cultivars in the control and simulated drought treatment groups (Fig. 4b).

Compared with their respective control groups, 11,375 DEGs were identified in JS6T6 (including 6553 up-regulated and 4822 down-regulated genes), 8762 DEGs were found in NM5T6 (including 4702 up-regulated and 4060 down-regulated genes), 4406 DEGs were noted in JS6T24 (including 2588 up-regulated and 1818 down-regulated genes), and 2512 DEGs were observed in NM5T24 (including 1069 up-regulated and 1443 down-regulated genes; Fig. 4a). Numerous DEGs were found in the T6 groups in both cultivars. DEGs in JS6T6 and JS6T24 treatments were approximately 0.298- and 0.754-fold higher than those in NM5T6 and NM5T24, respectively. Comparisons of GO categories and KEGG pathways between the cultivars showed that the DEGs in both cultivars were involved in similar GO categories and KEGG pathways (Additional file 1: Tables S4 and S5). On the basis of the Euler diagram, the comparisons of the control and T6 groups revealed that 1) 2334 genes (1207 up- and 1127 down-regulated genes) were specifically regulated in NM5, whereas 4947 genes (3071 up- and 1876 down-regulated genes), which were 1.12-fold more than that in NM5, were specifically regulated in JS6; 2) compared with their respective control groups, 3468 and 2919 genes were co-up- or co-down-regulated in both cultivars, respectively, whereas 14 and 27 genes were up- and down-regulated in JS6T6 but down- and up-regulated in NM5T6, respectively (Fig. 4c). The comparisons of the control and T24 groups revealed that 1) 1344 DEGs (558 up- and 786 down-regulated) were specifically regulated in NM5, whereas 3238 DEGs (1994 up- and 1244 down-regulated), which were 1.41-fold higher than those in NM5, were specifically regulated in JS6; 2) 488 and 551 genes were co-up- or co-down-regulated in both cultivars, respectively, whereas 106 and 23 genes were up- and down-regulated in JS6T24 but down- and up-regulated in NM5T24, respectively (Fig. 4d).

#### DEGs involved in oxidative stress responses

**Fig. 5:**
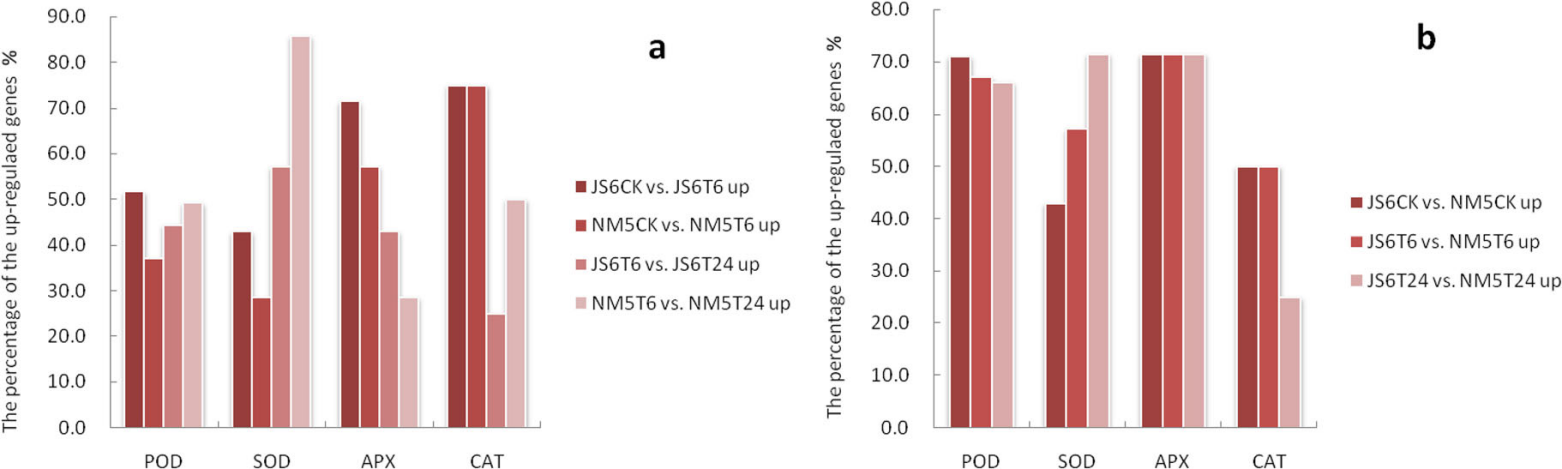
ROS scavenging enzyme-related genes in JS6 and NM5 differentially expressed in the presence and absence of simulated drought. DEGs were selected based on a cut-off of FDR ≤ 0.05 and |log2 FC| ≥ 1. **a** Percentages of these up-regulated DEGs from comparisons of JS6CK versus JS6T6, NM5CK versus NM5T6, JS6T6 versus JS6T24, and NM5T6 versus NM5T24. **b** Percentages of these up-regulated DEGs from comparisons of JS6CK versus NM5CK, JS6T6 versus NM5T6, and JS6T24 versus NM5T24

Compared with their respective controls, more genes belonging to POD, SOD, and APX families were induced in JS6 than in NM5 under T6 treatment, whereas more genes belonging to POD, SOD, and CAT families were induced in NM5 than in JS6 after simulated drought treatment from 6 h to 24 h (Fig. 5a). Specifically, regardless of the presence or absence of PEG-induced water stress, more genes belonging to POD and APX families exhibited higher expression levels in NM5 than in JS6. After simulated drought treatment, more genes belonging to SOD families showed higher expression levels in NM5 than in JS6 (Fig. 5b). Interestingly, more than 57–70% of the genes belonging to the APX family and 75% of genes belonging to the CAT family from both cultivars were up-regulated, whereas few genes belonging to the POD and SOD families from both cultivars were induced after PEG-induced water stress for 6 h. However, after PEG-induced water stress from 6 h to 24 h, an increase in the up-regulated genes belonging to the POD and SOD families and decrease in the up-regulated genes belonging to the APX and CAT families were observed (Fig. 5a).

#### DEGs involved in osmotic stress responses

**Fig. 6:**
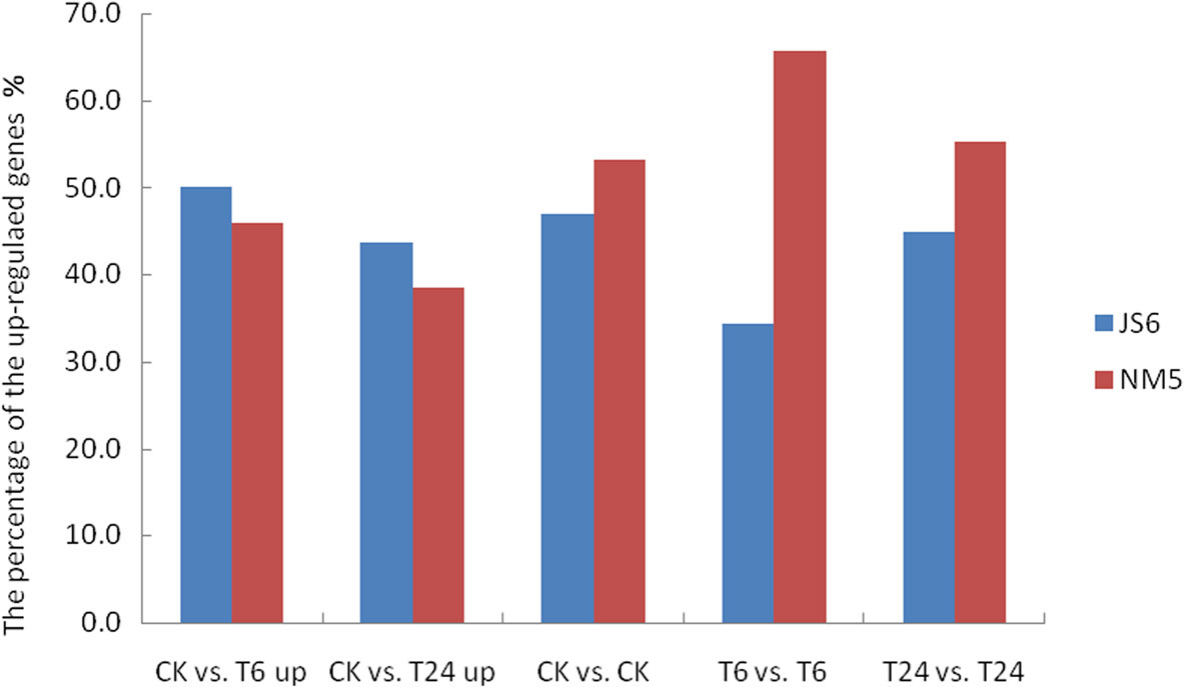
Proline-related genes in JS6 and NM5 differentially expressed in the presence and absence of simulated drought. DEGs were selected based on a cut-off of FDR ≤ 0.05 and |log2 FC| ≥ 1. Blocks from left to right indicate the percentages of these up-regulated DEGs from comparisons of JS6CK versus JS6T6, NM5CK versus NM5T6, JS6CK versus JS6T24, NM5CK versus NM5T24, JS6CK versus NM5CK, JS6T6 and NM5T6, and JS6T24 versus NM5T24

Regardless of the presence or absence of PEG-induced water stress, more genes related to proline biosynthesis maintained higher expression levels in NM5 than in JS6 (Fig. 6). However, compared with their respective controls, more genes involved in proline biosynthesis were up-regulated in JS6 than in NM5 under simulated drought treatment (Fig. 6).

#### DEGs involved in photosynthesis-associated responses

**Fig. 7:**
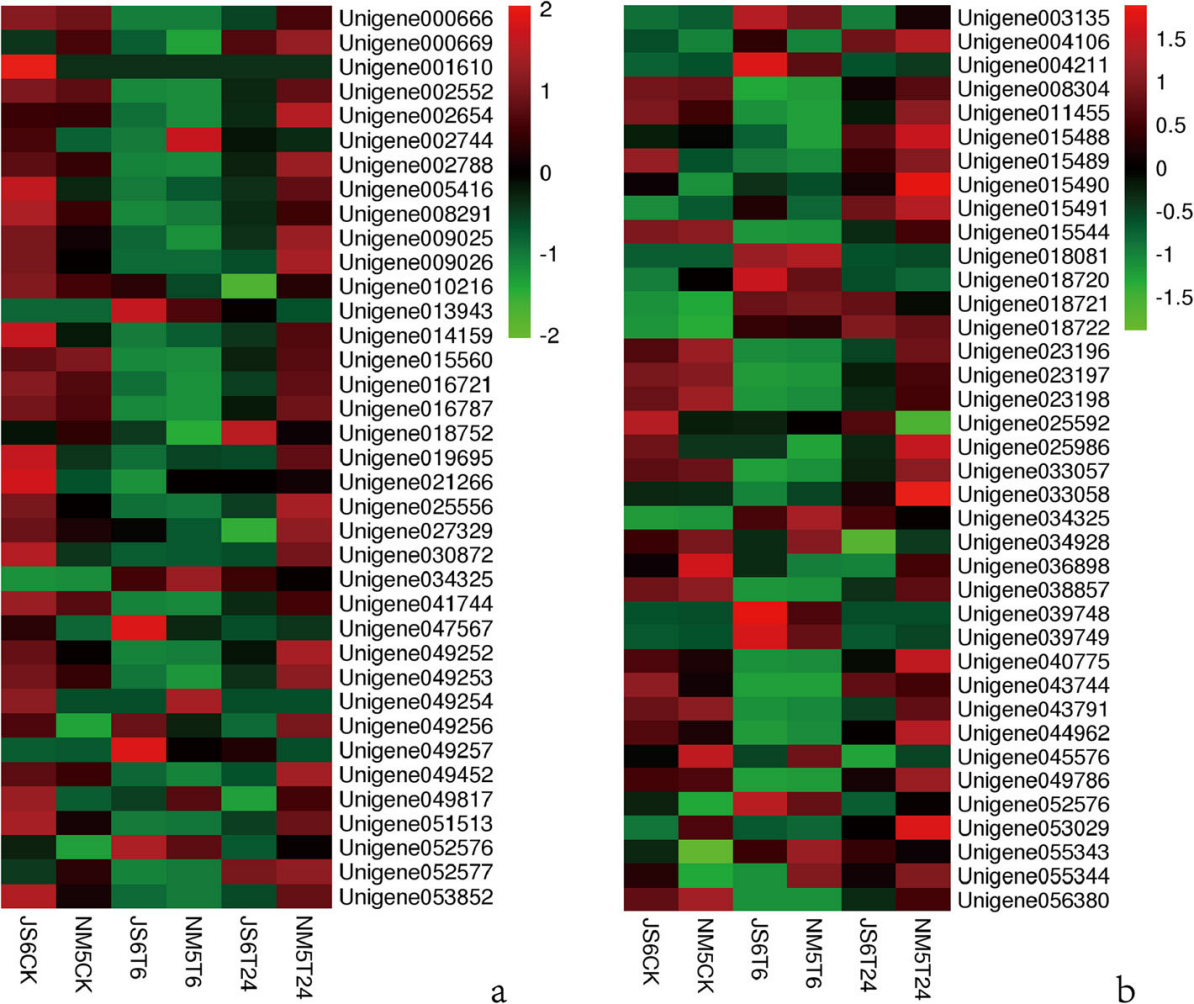
Comparisons of photosynthesis- (**a**) and chlorophyll-related genes (**b**) in JS6 and NM5 in the presence and absence of simulated drought. The heat map indicates the relative transcript levels of the genes

The photosynthesis-related genes were mostly suppressed under T6 and returned to being up-regulated after PEG-induced water stress from 6 h to 24 h in both cultivars (Fig. 7a). Compared with their respective controls, these genes were most repressed under T24 in JS6 but most induced under T24 in NM5. Compared with their respective control groups, 26 genes were co-down-regulated in both cultivars under T6 treatment, whereas four genes were down-regulated in JS6T6 but up-regulated in NM5T6. 25 genes were co-up-regulated after simulated drought treatment from 6 h to 24 h in both cultivars. 25 genes were down-regulated in JS6T24 but up-regulated in NM5T24 compared with their respective controls (Additional file 2: Figure S1a-c). Similar expression profiles were observed in genes related to chlorophyll content (Fig. 7b). Compared with their respective controls, 20 genes were co-down-regulated in both cultivars under T6 treatment, whereas four genes were down-regulated in JS6T6 but up-regulated in NM5T6. 23 genes were co-up-regulated after drought treatment from 6 h to 24 h in both cultivars. 14 genes were co-up-regulated in both cultivars under T24 treatment, whereas 11 genes were down-regulated in JS6T24 but up-regulated in NM5T24 compared with their respective controls (Additional file 2: Figure S2a-c).

### Expression patterns of the DEGs

**Fig. 8:**
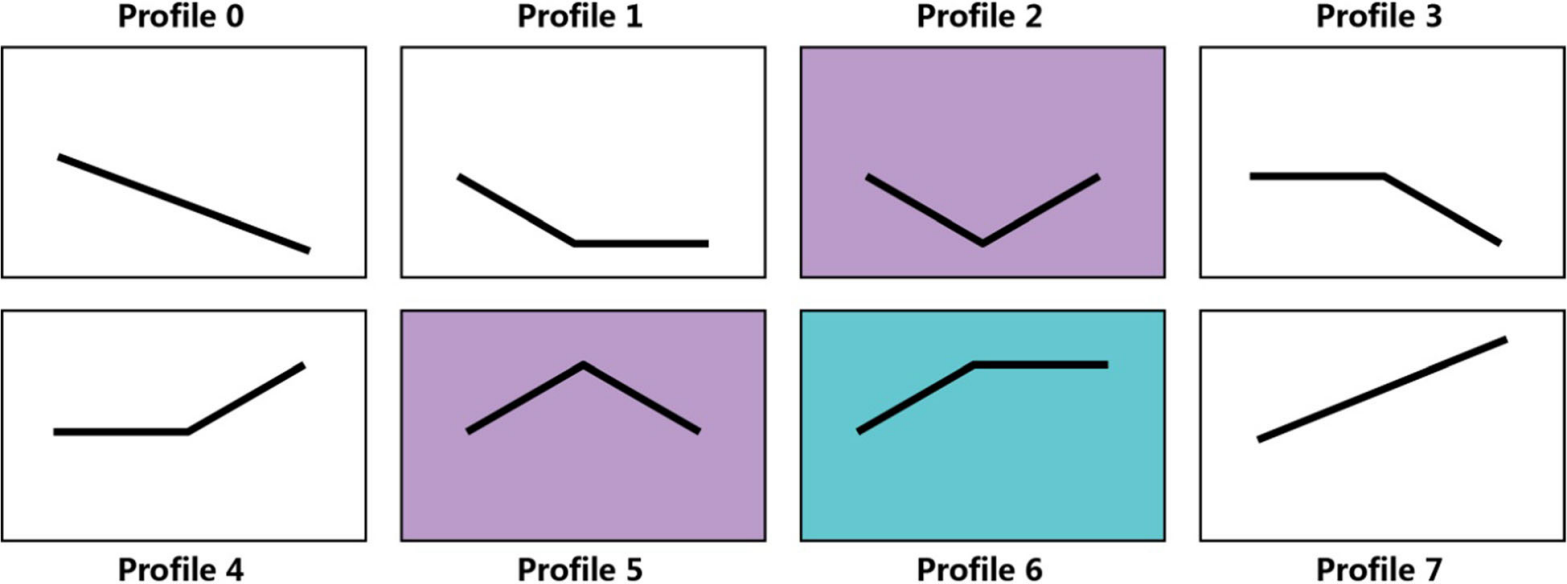
Expression patterns of the DEGs under the treatments in the two cultivars on the basis of STEM analysis

All these DEGs from each cultivar at different treatments were clustered separately in eight profiles (Fig. 8). In accordance with the profiles, considerable differences were observed in gene expression with stress time in response to PEG-induced water stress between the two cultivars. The DEGs were significantly enriched in profiles 2, 5, and 6 (*p* < 0.05) in JS6 but significantly enriched in profiles 2 and 5 (*p* < 0.05) in NM5. Profiles 2, 5, and 6 contained 4037, 3657, and 3329 DEGs in JS6, respectively, whereas profiles 2 and 5 included 4397 and 4615 DEGs in NM5, respectively.

The GO categories of “organic substance metabolic process”, “cellular metabolic process”, and “primary metabolic process” were enriched in the biological processes in both cultivars. “Membrane part” and “intrinsic component of membrane” were the most abundant categories in the cellular component, and genes involved in “organic cyclic compound binding” and “heterocyclic compound binding” were enriched in the molecular function of the cultivars (Additional file 1: Table S6).

KEGG pathway enrichment analysis annotated 1943 and 1738 DEGs from JS6 and NM5, respectively. Some important KEGG pathways, including the top 10 pathways with the highest quantities of DEGs and the photosynthesis pathway, are shown in Additional file 1: Table S7. These pathways included plant hormone signal transduction, carbon metabolism, ribosome, phenylpropanoid biosynthesis, biosynthesis of amino acids, starch and sucrose metabolism, protein processing in endoplasmic reticulum, plant–pathogen interaction, and spliceosome and purine metabolism, or glycolysis/gluconeogenesis.

#### Expression patterns of the DEGs involved in abiotic and biotic stress response-related biological processes

**Fig. 9:**
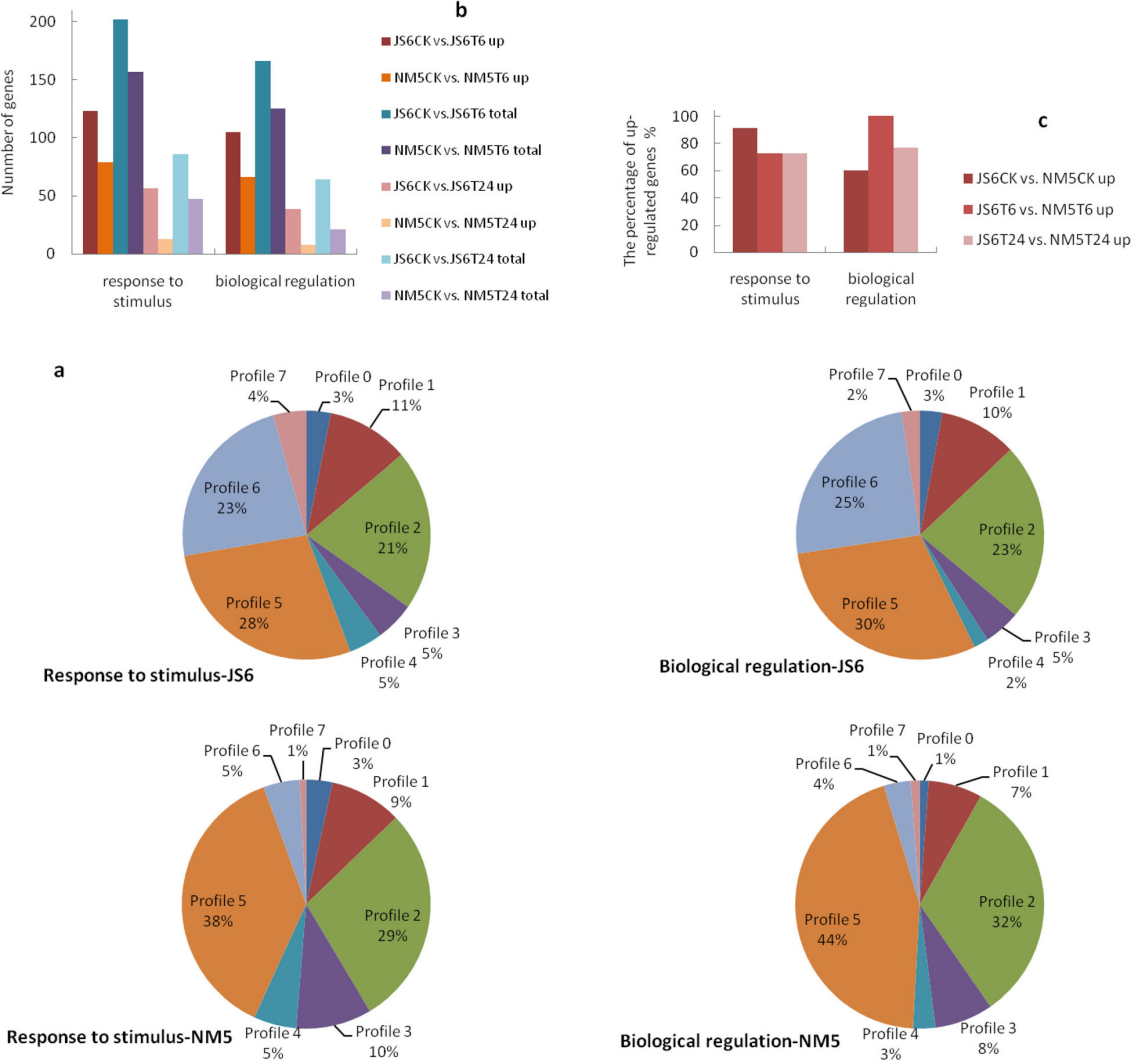
Genes involved in abiotic and biotic stress response-related biological processes in JS6 and NM5 differentially expressed in the presence and absence of simulated drought. DEGs were selected based on a cut-off of FDR ≤ 0.05 and |log2 FC| ≥ 1. **a** Expression patterns of these DEGs under the treatments in the two cultivars. **b** Comparisons of these DEGs drawn from JS6CK versus JS6T6, NM5CK versus NM5T6, JS6CK versus JS6T24, and NM5CK versus NM5T24. **c** Comparisons of these up-regulated DEGs drawn from JS6CK versus NM5CK, JS6T6 versus NM5T6, and JS6T24 versus NM5T24

Genes involved in “response to stimulus” were enriched in profiles 5 and 6 in JS6, whereas these genes were enriched in profile 5 in NM5 (Fig. 9a). Similar findings were observed in the GO categories of “biological regulation” in both cultivars (Fig. 9a). The main expression patterns of these genes showed that they were promoted at T6 but suppressed at T24 (profile 5) or maintained high expression level at T24 (profile 6) in JS6. By contrast, the promotion of these genes at T6 and the repression at T24 (profile 5), which is the main expression pattern, were observed in NM5.

Compared with their respective controls, approximately 28.7% more DEGs related to “response to stimulus” were identified in JS6T6 than in NM5T6, including 55.7% more up-regulated genes. Moreover, 32.8% more DEGs related to “biological regulation” were observed in JS6T6 than in NM5T6, including 59.1% more up-regulated genes. Approximately 83.0% more DEGs related to “response to stimulus” were identified in JS6T24 than in NM5T24, including approximately 3.31-fold more up-regulated genes. More than twice more DEGs related to “biological regulation” were identified in JS6T24 than in NM5T24, including approximately 3.88-fold more up-regulated genes (Fig. 9b). However, more genes in NM5 involved in “response to stimulus” and “biological regulation” showed higher expression levels than those in JS6, regardless of the presence or absence of PEG-induced water stress (Fig. 9c).

#### Expression patterns of the DEGs involved in plant hormone signal transduction and photosynthesis pathways

**Fig. 10:**
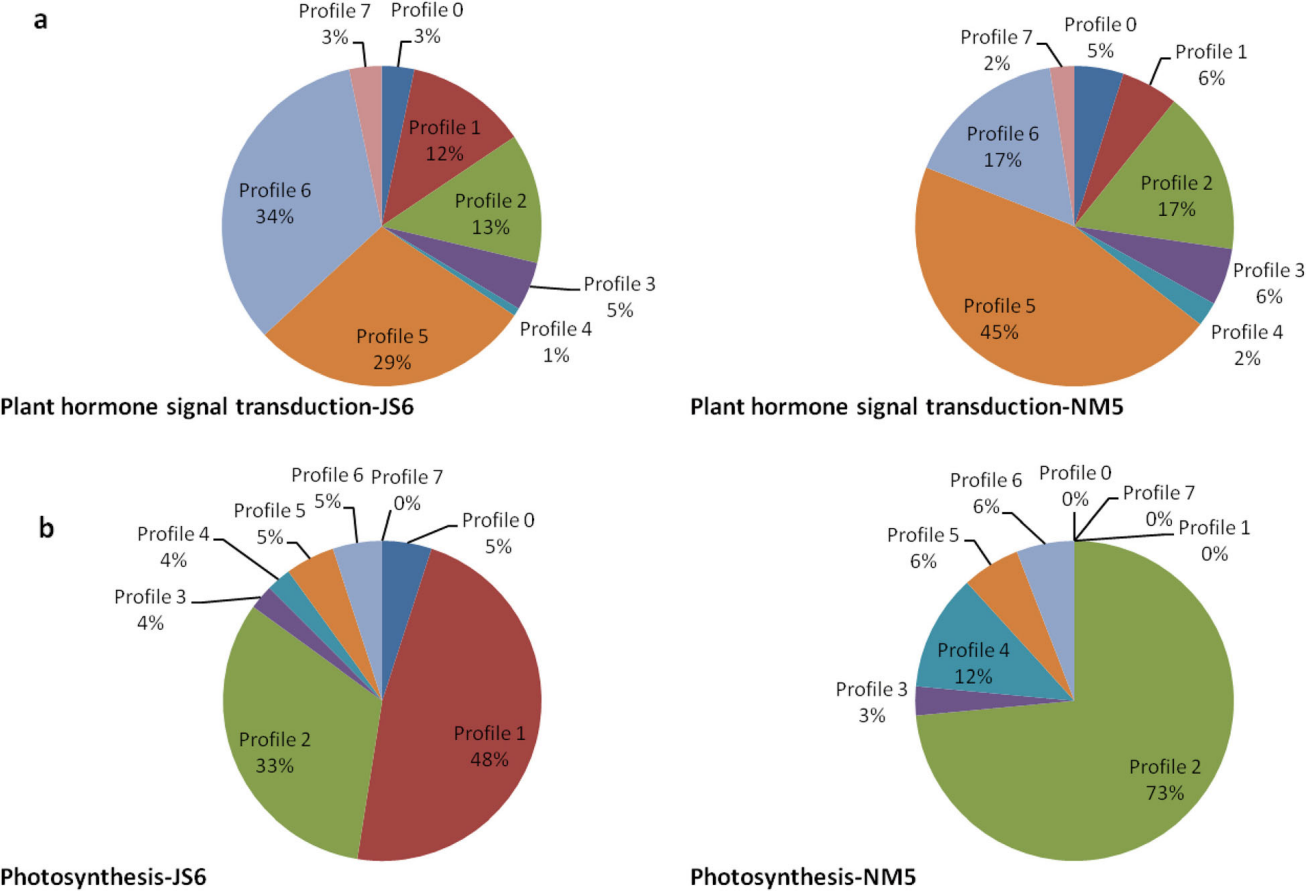
Genes involved in plant hormone signal transduction and photosynthesis pathways in JS6 and NM5 differentially expressed in the presence and absence of simulated drought. DEGs were selected based on a cut-off of FDR ≤ 0.05 and |log2 FC| ≥ 1. **a** Expression patterns of the DEGs involved in the plant hormone signal transduction pathway under the treatments in the two cultivars. **b** Expression patterns of the DEGs involved in the photosynthesis pathway under the treatments in the two cultivars

A total of 122 and 121 DEGs were annotated to plant hormone signal transduction pathways in JS6 and NM5, respectively, and these items showed the highest representations. About 45% (55/121) of the genes were enriched in profile 5 in NM5, which accounted for the largest part; however, 34% (41/122) of the genes were enriched in profile 6 and 29% (35/122) were enriched in profile 5 in JS6, accounting for the largest and second largest parts in JS6 (Fig. 10a), respectively. Moreover, 40 and 34 DEGs were annotated to the photosynthesis pathways in JS6 and NM5, respectively. Approximately 73% (25/34) of the genes were enriched in profile 2 in NM5, which accounted for the largest part, and none of the genes belonged to profile 1 in NM5. However, 48% (19/40) of the genes were enriched in profile 1, and 33% (13/40) of the genes were enriched in profile 2 in JS6, which accounted for the largest and second largest parts in JS6 (Fig. 10b), respectively.

**Fig. 11:**
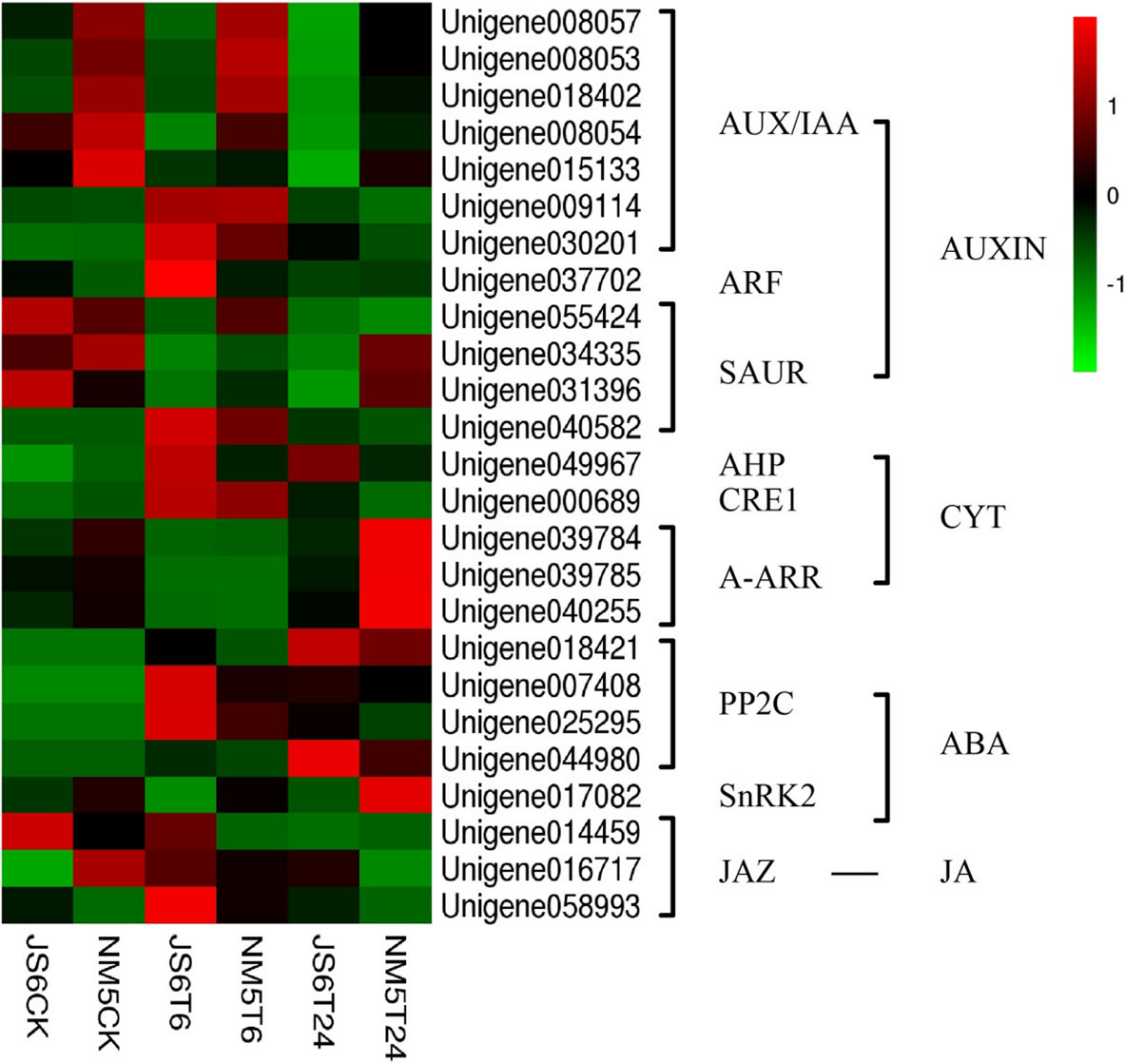
Comparisons of the genes involved in the plant hormone signal transduction pathways in JS6 and NM5 in the presence and absence of simulated drought treatments. The heat map indicates the relative transcript levels of the genes

We focused on two pairwise comparisons, namely, JS6T6 versus NM5T6 and JS6T24 versus NM5T24, in the analysis of plant hormone signal transduction pathways (Fig. 11). Compared with JS6, four unigenes encoding auxin-induced protein AUX/IAA were up-regulated under T6, four unigenes were up-regulated under T24, and two unigenes were down-regulated under T24 in NM5. A repressed unigene encoding ARF and an up-regulated unigene encoding SAUR were found in NM5T6, whereas two up-regulated and one down-regulated unigenes involved in SAUR encoding were identified in NM5T24. *Unigene 049967* encoding AHP and *Unigene 000689* encoding CRE1 were suppressed in NM5T6 and NM5T24, respectively. *Unigene039784*, *Unigene039785*, and *Unigene040255* encoding A-ARR were up-regulated under T24 in NM5. Two of the unigenes involved in encoding PP2C were down-regulated under T6 in NM5, and two other unigenes were suppressed under T24 in NM5. *Unigene 014459* and *Unigene 058993* involved in the jasmonate ZIM domain proteins (JAZ) encoding were repressed under T6 in NM5. Moreover, *Unigene 016717* and *Unigene 058993* were suppressed under T24 in NM5.

### qRT-PCR validation

**Fig. 12:**
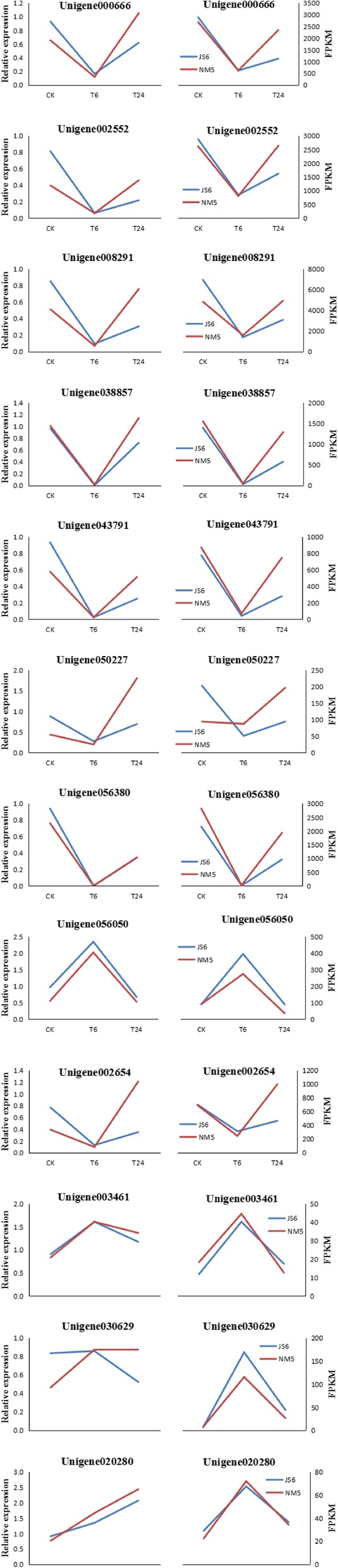
Expression patterns of twelve candidate transcripts measured in JS6 and NM5 via qRT-PCR (left side) and RNA-seq (right side). CK, T6, and T24 represent different treatments using Hoagland solution containing 20% PEG-6000 for 0, 6, and 24 h, respectively

**Fig. 13:**
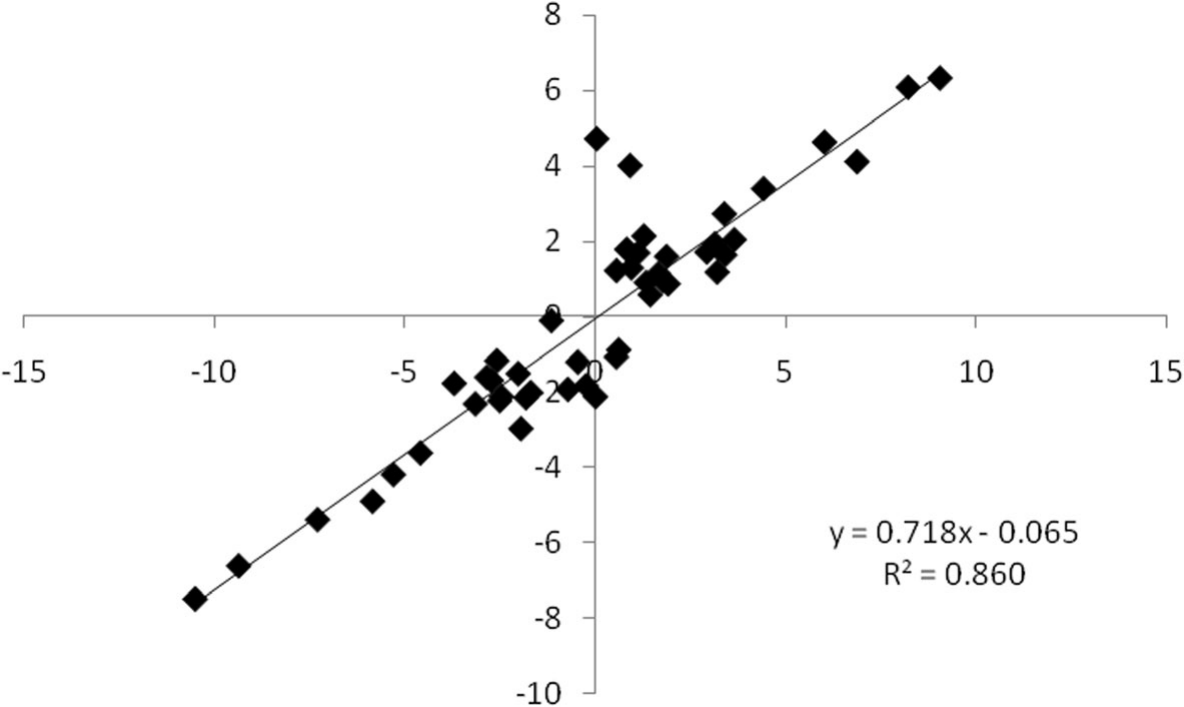
Correlation between qRT-PCR and RNA-seq based on their respective data from the twelve candidate transcripts. Each point represents a fold change value of expression at T6 or T24 compared with that of CK

Both qRT-PCR and RNA-seq data demonstrated that the expression levels of the eight transcripts, namely, *Unigene 000666*, *Unigene 002552*, *Unigene 002654*, *Unigene 008291*, *Unigene 038857*, *Unigene 043791*, *Unigene 050227*, and *Unigene 056380*, were repressed at T6 and promoted at T24 (Fig. 12); by contrast, *Unigene 003461* and *Unigene 056050* were up-regulated at T6 but down-regulated at T24. The expression patterns of these transcripts obtained by qRT-PCR were highly consistent with the RNA-seq results (Figs. 12 and 13). A significantly positive correlation between the RNA-seq and qRT-PCR data was revealed via linear regression analysis (Fig. 13), thereby confirming the DEGs data from RNA-seq.

## Discussion

Diverse cultivars with different genetic backgrounds provide important genetic resources to breed new varieties with improved traits [46]. Proso millet is highly adaptive to drought stress, and studying its responses to drought stress may provide important information on the mechanisms underlying its drought tolerance, as well as improve its drought adaptation and that of other crops. In this study, two proso millet cultivars, NM5 and JS6, and the responses of their leaves to drought were analyzed and discussed.

### Overall analysis of transcriptional information of JS6 and NM5 cultivars

From the RNA-seq data, 115,660 transcripts and 59,035 unigenes were obtained with mean lengths of 1367 and 1080 bp, respectively. Among them, 33,634 unigenes were successfully annotated in at least one of the four public databases, namely, nr, SWISS-PROT, COG, and KEGG, using the BLASTX algorithm. These data will further enrich the transcriptome information of proso millet.

When plants experience drought stress, their gene expression levels commonly show extensive changes [47]. In the present study, in response to PEG-induced water stress, drastic transcriptional changes occurred at T6 in both cultivars and more DEGs were observed in the sensitive cultivar than in the tolerant one. More DEGs were identified in T6 treatments than in T24 treatments, compared with their respective controls in both cultivars, whereas the number of DEGs between JS6T6 and NM5T6 was found to be the lowest (Fig. 4a). These results suggested that increased transcriptional changes occurred at T6 in both cultivars, and the drought-tolerant cultivar may maintain highly stable gene expression levels during drought stress. Yates et al. [48] reported similar findings and attributed their results to the different responses in drought-sensitive and drought-tolerant plants; this characteristic may be caused by the different sensitivities of the two plants to drought.

In response to PEG-induced water stress, the sensitive cultivar exhibited more DEGs, especially up-regulated genes, which were involved in regulating responses to stimulus and biological regulation processes, than the drought-tolerant cultivar (Fig. 9b). However, more of these genes maintained higher transcript levels in the drought-tolerant cultivar, regardless of the presence or absence of simulated drought treatment (Fig. 9c). Under drought conditions, the drought-tolerant cultivar exhibited a lower requirement of intensifying the regulation of relevant genes than the sensitive cultivar. This phenomenon may be due to the less severe conditions and higher transcript levels of relevant genes in the drought-tolerant cultivar compared with the sensitive cultivar.

### Transcriptional changes related to plant photosynthesis

In the presence of simulated drought stress, the genes related to plant photosynthesis were most suppressed under T6 (Fig. 7a). Similar expression profiles were found in the genes related to the chlorophyll content (Fig. 7b). These findings agreed with the characteristics of plant photosynthesis in the presence of drought stress. Previous studies have demonstrated that a plant’s photosynthetic activity is reduced under drought stress [33, 49, 50], and photosynthesis-related genes are preferentially repressed under drought stress [17, 48]. Notably, qRT-PCR data also presented similar characteristics. On the basis of functional annotation, *Unigene 000666* was predicted to encode subunit O in photosystem I, which was predicted to be related to the intrinsic component of the membrane. *Unigene 008291* and *Unigene 002552* putatively encode subunits II and III in the photosystem I reaction center in the chloroplast, respectively, and both were predicted to be related to cellular metabolic processes. *Unigene002654* was predicted to encode a 22 kD protein in the photosystem II reaction center in chloroplast, which was predicted to be related to energy metabolic processes. *Unigene 038857*, *Unigene 043791,* and *Unigene 056380* were predicted to encode CP26 precursor, chlorophyll a-b binding apoprotein, and CP24 and P4 chlorophyll a-b binding protein, respectively, which were predicted to contribute to important metabolic and protein-modification processes. *Unigene 050227* putatively encoded the photosynthetic NDH subunit of lumenal location 2 in the chloroplast. The tested genes related to plant photosynthesis were all repressed at T6 and promoted at T24 (Fig. 12).

The genes related to plant photosynthesis were most recovered and up-regulated after drought treatment from 6 h to 24 h in both cultivars. However, the genes were most repressed under T24 in JS6 and most induced under T24 in NM5 compared with their respective controls (Additional file 2: Figure S1c). The results of KEGG pathway enrichment analysis and qRT-PCR data also produced similar characteristics (Figs. 10b and 12). The photosynthesis-related genes that were repressed under drought stress could more easily return to relatively normal levels in the drought-tolerant cultivar than in the drought-sensitive cultivar. Previous studies have also demonstrated that more suppressed genes related to the photosynthetic apparatus are found in the sensitive pool than in the tolerant one [48], possibly because drought stress reduces the photosynthetic capacity of plants. Improved strategies on regulating drought tolerance in drought-tolerant cultivars, including increased expression levels of the genes related to ROS scavengers and proline accumulation, help the plants reduce ROS injury, survive drought stress, and maintain productivity. Photosynthetic recovery contributes to plant recovery after drought [51]. Thus, when stress injury is mitigated after a series of defence responses, drought-tolerant cultivars recover more easily than drought-sensitive cultivars, which maintain large portions of their photosynthesis apparatus. Drought stress would have lower adverse effects on crop growth and yield of tolerant cultivars than on those of sensitive ones.

### Changes in proso millet cultivars in response to oxidative stress

Drought treatment increases MDA accumulation [52–54], and drought-sensitive cultivars accumulate more MDA than tolerant ones [54, 55]. The present study showed that the MDA contents increased in both cultivars as the stress time continued, and the MDA content in JS6 was consistently higher than that in NM5, regardless of the presence or absence of PEG-induced water stress (Fig. 1). The results indicated that the drought-sensitive cultivar JS6 suffered higher levels of ROS injury than the drought-tolerant one under PEG-induced water stress. Consistent with these results, more genes with high transcript levels that were predicted to be related to ROS scavenging enzyme in the drought-tolerant cultivar (Fig. 5b) were identified in this study. Regardless of the presence or absence of PEG-induced water stress, more genes related to proline biosynthesis maintained higher transcript levels in the tolerant cultivar than in the sensitive one (Fig. 6), thereby contributing to the low levels of ROS injury in the drought-tolerant cultivar. During osmotic stress, proline accumulation could protect proteins and membranes by protecting the enzymes of the glutathione–ascorbate cycle or enhancing their activities [2]. Thus, in response to drought stress, tolerant cultivars were more effective than sensitive cultivars in protecting the membrane system from the synergistic effects of ROS scavenging and osmotic regulation systems, regulating ROS homeostasis, reducing ROS injury, and improving drought tolerance.

In the presence of simulated drought stress, APXs and CATs showed faster transcriptional responses than PODs and SODs, and the drought-responsive genes involved in ROS scavenging systems in the drought-tolerant cultivar showed delayed transcriptional responses compared with those in the sensitive cultivar. The synergistic effects and non-synchronization of the ROS scavenging system have been reported in previous studies on maize [56] and rice [57]. ROS scavenging enzyme activity in the drought-tolerant cultivars was higher than that in sensitive ones [57]. These findings are helpful to further elucidate the mechanisms of the response of proso millet to future systematic study on changes in the proline content and ROS scavengers in the presence of drought stress among different drought-tolerant cultivars.

### Effects of plant hormones on drought tolerance and growth of proso millet

Compared with the drought-sensitive cultivar JS6, JAZ was suppressed in the JA signalling pathways in the tolerant cultivar NM5 under simulated drought stress. Under drought stress, the accumulation of active JA was probably higher in the drought-tolerant cultivar, which repressed JAZ and facilitated the JA signal transduction pathway, than in the sensitive cultivar. JA is important in the response to environmental stimuli [22–24]. It can promote ROS detoxification enzyme systems and ascorbic-recycling genes [26]. The abundance of ascorbate is implicated in the activation threshold of hormone signals and in hormone cross-talk [29]. JAZ can repress the expression of JA-responsive genes by suppressing the transcription factor in plant cells with low JA levels [58]. Under environmental stimuli, the accumulation of active JA stimulates JAZ degradation via the ubiquitin/26S proteasome pathway to release the transcription factors from repression and trigger JA signalling pathways [59]. Thus, in the presence of drought, the different response strategies of the JA signal transduction pathway in the drought-tolerant cultivar may be one of the driving forces underlying drought stress tolerance. During drought stress, the high accumulation of active JA in the drought-tolerant cultivar possibly facilitated the JA signal transduction pathway, promoted ROS scavenging system, reduced ROS injury, and elevated cell membrane stability, all of which mitigated the stress conditions and made photosynthetic recovery easy.

Compared with JS6, auxin and cytokinin in the signal transduction pathways were repressed under T6 in NM5. This reaction was helpful to regulate stomatal closure in the drought-tolerant cultivar under stress treatment. Moreover, cytokinin in the signal transduction pathways was induced under T24 in NM5. This finding agreed with the hypotheses that drought-tolerant cultivars possess greater recovery capacities after a series of defence responses compared with sensitive cultivars. Furthermore, the JA and ABA signal transduction pathways were promoted under each stress treatment in NM5, which helped regulate stomatal closure and activate downstream defence responses during stress treatments. A further systematic study on the changes in the plant hormones of different drought-tolerant cultivars during response to drought will provide important information to further elucidate the mechanisms of the response of proso millet.

## Conclusion

In the presence of simulated drought stress, NM5 maintained highly stable gene expression levels and carried more genes with high transcript levels that were predicted to be related to the ROS scavenging enzyme compared with JS6. Compared with drought-sensitive cultivars, the different regulation strategies of the JA signal transduction pathway in drought-tolerant cultivars may be one of the driving forces of drought stress tolerance.

## Methods

### Plant materials and drought treatment

In this study, PEG-induced osmotic stress was used to simulate drought stress. On the basis of germination rates, drought-sensitive and drought-tolerant materials for subsequent analysis were selected among 37 core Chinese proso millet cultivars. All the above-mentioned proso millet cultivars have been released in China and all the test materials were provided by the Minor Grain Research Group of the College of Agronomy, Northwest A & F University from cultivated plants in the fields.

A total of 600 seeds of similar sizes and appearance were selected from each proso millet cultivar and randomly divided into six groups for germination. All seeds were pre-processed via oven drying at 30 °C–35 °C for 48–72 h. Subsequently, 100 seeds from each cultivar were distributed in a plastic box (12 cm × 12 cm × 8 cm) with filter paper and treated with 8 mL of distilled water as the control and 8 mL of 20% PEG 6000 solution as the intervention. Finally, seeds were grown in a covered plastic box under a photoperiod of 0 h light/24 h dark and humidity of 70% at 25 °C for 3 days. The germination rates were then measured. The cultivars in the control group with germination rates higher than 95% and those in the treatment group with the highest or lowest relative germination rates were selected as the drought-tolerant and drought-sensitive cultivars, respectively, for subsequent analysis.

Seeds of both cultivars were germinated and grown in distilled water in plastic boxes for 3 days under a photoperiod of 0 h light/24 h dark and humidity of 70% at 25 °C. The plants were cultivated in Hoagland solution under a photoperiod of 16 h light/8 h dark and humidity of 70% at 25 °C. At the three-leaf stage, the seedlings of both cultivars were treated for 0 (CK), 6 (T6), and 24 h (T24) with Hoagland solution containing 20% PEG-6000. After the treatments, leaf tissues from each group were sampled for subsequent analysis, rapidly frozen in liquid nitrogen, and stored at − 80 °C.

### Measurements of MDA, RNA extraction, transcriptome sequencing, and de novo sequence assembly

MDA levels were measured as described previously [60]. Total RNA from the collected leaf tissues was extracted using TRIzol® reagent (Invitrogen) in accordance with the manufacturer’s instructions. The integrity of the extracted RNAs was assessed via agarose gel electrophoresis using Agilent 2100 Bioanalyzer (AgilentTechnologies, Palo Alto, CA, USA). The quantity was determined with a NanoDrop 8000 spectrophotometer (NanoDrop, Wilmington, DE, USA). The quality of all the extracted RNAs was accepted for library construction containing total RNA with RIN values > 6.3 and 28S:18S ratios ≥1.1.

Total mRNAs were enriched by using oligo (dT) magnetic beads and further sheared into short fragments by adding a fragmentation buffer. These RNA fragments were involved in the synthesis of first-strand cDNA with random hexamers, followed by the synthesis of second-strand cDNA with DNA polymerase I and RNase H (Invitrogen). After purification using a QiaQuick PCR extraction kit (QIAGEN), end-repaired and A-tailed sequencing adaptors were ligated onto the cDNA fragments. Suitable fragments were purified using AMPure XP beads and amplified via PCR amplification to construct a library for subsequent sequencing. Transcriptome sequencing was performed on the HiSeq™ 4000 platform (SageneBiotech Co. Ltd., Guangzhou, China). The raw image data from sequencing were transformed into raw reads via base calling. High-quality clean reads were obtained by strict filtering steps, including removal of adapter sequences, reads containing more than 10% unknown bases, and low-quality reads. The obtained high-quality clean reads were mixed and assembled using Trinity program.

### Functional annotation and DEG analysis

To determine the putative function, we searched the assembled unigenes against the databases, including the nr (http://www.ncbi.nlm.nih.gov/), SWISS-PROT (http://www.expasy.ch/sprot/), KEGG (http://www.genome.jp/kegg/), and KOG (http://www.ncbi.nlm.nih.gov/cog/) databases by using the BLASTX algorithm with an *e*-value of <1e^− 5^. The assembled unigenes were annotated with the GO database by the Blast2GO program based on NR annotation, and GO functional classification of unigenes was performed using WEGO.

The expression values of reads were normalized with Reads Per Kilobase of exon Model per Million mapped reads. The threshold *P* was adjusted using the false discovery rate (FDR) in multiple hypothesis testing. In this study, the cut-off was set as FDR ≤ 0.05 and |log2 FC| ≥ 1 to determine the DEGs using edgeR package (http://www.r-project.org/). All the DEGs were subjected to enrichment analysis of GO functions and KEGG pathways.

### Quantitative real-time PCR analysis

Additional file 1: Table S1 shows the sequence of the primers involved in the qRT-PCR experiments. SYBR premix Ex Taq kit (TaKaRa, Dalian, China) was used in the experiment, and qRT-PCR was performed on an ABI 7500 Real-Time System (Applied Biosystems) with first-strand cDNA as the template. To confirm the DEG results, 12 transcripts were randomly selected for qRT-PCR validation. Actin served as the internal control. The fold change in the expression levels of target genes was calculated via relative quantification (2^-△△CT^) [13].

## Additional files

Additional file 1: **Table S1.** Information on the primers involved in the qRT-PCR experiments. **Table S2.** Germination rates of 37 core Chinese proso millet cultivars. **Table S3.** Information on the pathways with the most quantities of DEGs in different KEGG classes. **Table S4.** Information on the top 30 GO categories with the most quantities of DEGs in the three major GO classes in both cultivars. **Table S5.** Information on the DEGs involved in the KEGG pathways in both cultivars. **Table S6.** Level 3 GO annotations of the DEGs in three major GO classes in both cultivars. **Table S7.** Profiles of the top 10 pathways with the most quantities of DEGs and photosynthesis pathway based on KEGG pathway enrichment analysis. (XLSX 36 kb)

Additional file 2: JS6 and NM5 were treated with 20% PEG-6000 solution for 6 (JS6T6 and NM5T6, respectively) and 24 h (JS6T24 and NM5T24, respectively) or with water (control group; JS6CK and NM5CK, respectively). **Figure S1.** DEGs involved in photosynthesis in JS6 and NM5 in the presence and absence of drought treatments. **a** Euler diagram of the DEGs involved in photosynthesis from comparisons between the control and T6 treatment groups, including up- and down-regulated genes in JS6T6 and NM5T6. **b** Euler diagram of the DEGs involved in photosynthesis from comparisons between the T6 and T24 treatment groups, including up- and down-regulated genes. **c** Euler diagram of the DEGs involved in photosynthesis from comparisons between the control and T24 treatment groups, including up- and down-regulated genes in JS6T24 and NM5T24. **Figure S2.** Chlorophyll content-related genes in JS6 and NM5 differentially expressed in the presence and absence of drought treatments. **a** Euler diagram of the DEGs related to chlorophyll from comparisons between the control and T6 treatment groups, including up- and down-regulated genes in JS6T6 and NM5T6. **b** Euler diagram of the DEGs related to chlorophyll from comparisons between T6 and T24 treatment groups, including up- and down-regulated genes. **c** Euler diagram of the DEGs related to chlorophyll from comparisons between the control and T24 treatment groups, including up- and down-regulated genes in JS6T24 and NM5T24. (ZIP 544 kb)

## Data Availability

All the raw reads of RNA sequencing data were deposited in the NCBI Short Read Archive (SRA) database under the accession number SRP144636 (https://www.ncbi.nlm.nih.gov/sra/SRP144636), as associated with the BioProject PRJNA454008. The transcriptome shotgun assembly data were deposited at DDBJ/EMBL/GenBank under the accession number GHHA01000000.

## Abbreviations

ABA: Abscisic acid
BLAST: Basic local alignment search tool
CAT: Catalase
cDNA: Complementary deoxyribonucleic acid
DEGs: Differentially expressed genes
FC: Fold change
FDR: False discovery rate
FPKM: Fragments per kilobase of exon per million fragments mapped
GO: Gene Ontology
JA: Jasmonic acid
JAZ: Jasmonate ZIM domain proteins
MDA: Malondialdehyde
POD: Peroxidase
qRT-PCR: Quantitative real-time polymerase chain reaction
RNA-seq: RNA sequencing
ROS: Reactive oxygen species
SOD: Superoxide dismutase
