# Supplementary figures and images for: Comparative analysis of proso millet (Panicum miliaceum L.) leaf transcriptomes for insight into drought tolerance mechanisms
Source: BMC Plant Biol. 2019 Sep 11;19:397. doi: 10.1186/s12870-019-2001-x (PMC6737659; doi:10.1186/s12870-019-2001-x)

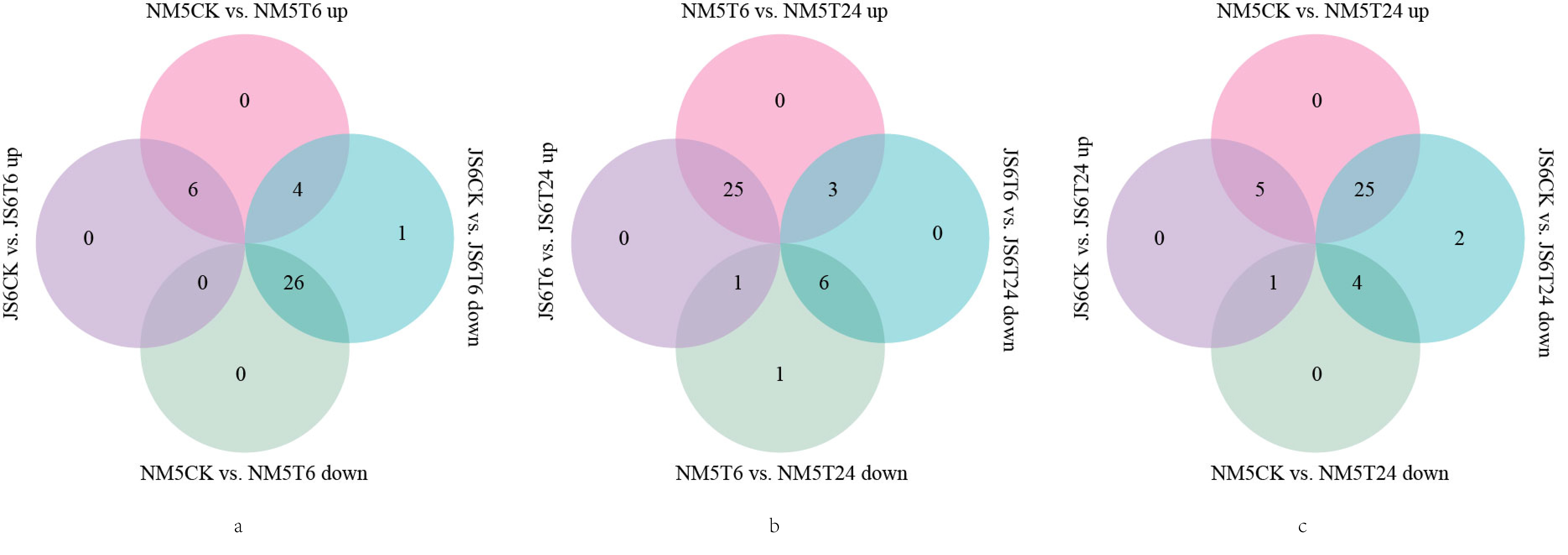

Supplement: Supplementary file 2 — JS6 and NM5 were treated with 20% PEG-6000 solution for 6 (JS6T6 and NM5T6, respectively) and 24 h (JS6T24 and NM5T24, respectively) or with water (control group; JS6CK and NM5CK, respectively). Figure S1. DEGs involved in photosynthesis in JS6 and NM5 in the presence and absence of drought treatments. a Euler diagram of the DEGs involved in photosynthesis from comparisons between the control and T6 treatment groups, including up- and down-regulated genes in JS6T6 and NM5T6. b Euler diagram of the DEGs involved in photosynthesis from comparisons between the T6 and T24 treatment groups, including up- and down-regulated genes. c Euler diagram of the DEGs involved in photosynthesis from comparisons between the control and T24 treatment groups, including up- and down-regulated genes in JS6T24 and NM5T24. Figure S2. Chlorophyll content-related genes in JS6 and NM5 differentially expressed in the presence and absence of drought treatments. a Euler diagram of the DEGs related to chlorophyll from comparisons between the control and T6 treatment groups, including up- and down-regulated genes in JS6T6 and NM5T6. b Euler diagram of the DEGs related to chlorophyll from comparisons between T6 and T24 treatment groups, including up- and down-regulated genes. c Euler diagram of the DEGs related to chlorophyll from comparisons between the control and T24 treatment groups, including up- and down-regulated genes in JS6T24 and NM5T24. (ZIP 544 kb) [file 12870_2019_2001_MOESM2_ESM.zip › Fig S1.jpg]

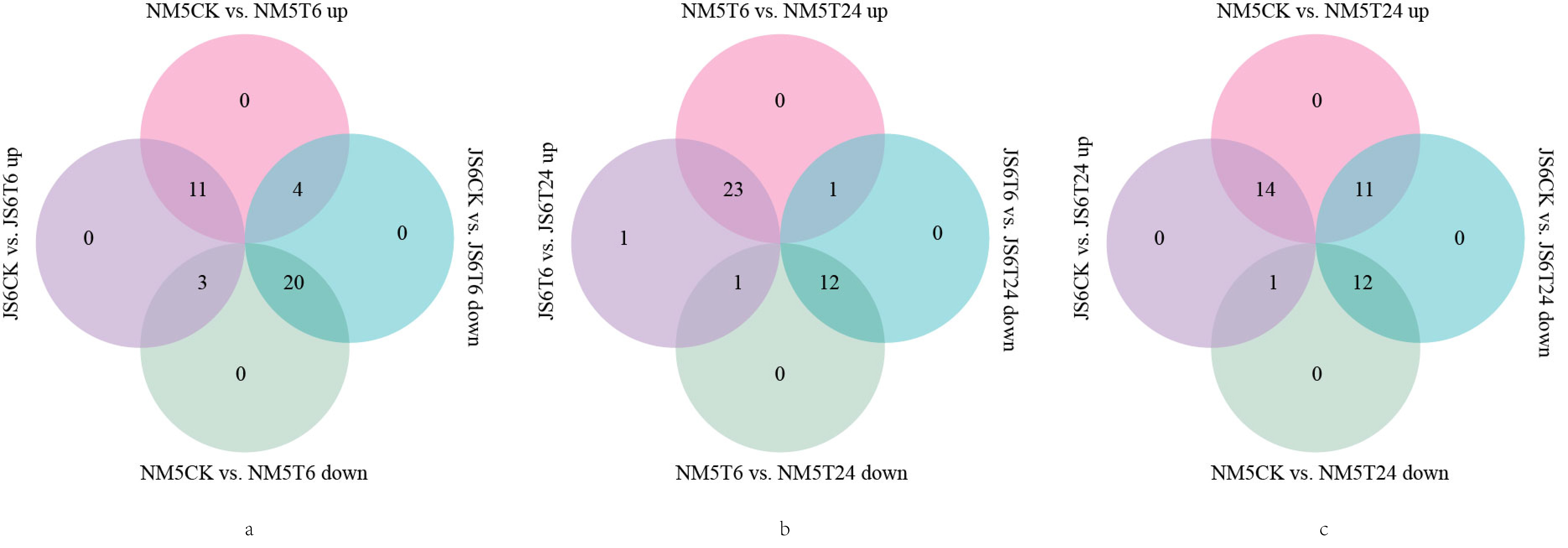

Supplement: Supplementary file 2 — JS6 and NM5 were treated with 20% PEG-6000 solution for 6 (JS6T6 and NM5T6, respectively) and 24 h (JS6T24 and NM5T24, respectively) or with water (control group; JS6CK and NM5CK, respectively). Figure S1. DEGs involved in photosynthesis in JS6 and NM5 in the presence and absence of drought treatments. a Euler diagram of the DEGs involved in photosynthesis from comparisons between the control and T6 treatment groups, including up- and down-regulated genes in JS6T6 and NM5T6. b Euler diagram of the DEGs involved in photosynthesis from comparisons between the T6 and T24 treatment groups, including up- and down-regulated genes. c Euler diagram of the DEGs involved in photosynthesis from comparisons between the control and T24 treatment groups, including up- and down-regulated genes in JS6T24 and NM5T24. Figure S2. Chlorophyll content-related genes in JS6 and NM5 differentially expressed in the presence and absence of drought treatments. a Euler diagram of the DEGs related to chlorophyll from comparisons between the control and T6 treatment groups, including up- and down-regulated genes in JS6T6 and NM5T6. b Euler diagram of the DEGs related to chlorophyll from comparisons between T6 and T24 treatment groups, including up- and down-regulated genes. c Euler diagram of the DEGs related to chlorophyll from comparisons between the control and T24 treatment groups, including up- and down-regulated genes in JS6T24 and NM5T24. (ZIP 544 kb) [file 12870_2019_2001_MOESM2_ESM.zip › Fig S2.jpg]
